# Development of an Experimental Setup and a Validated Model for a Highly Integrated Biocatalytic Process

**DOI:** 10.1021/cbe.5c00167

**Published:** 2026-03-26

**Authors:** Francesca von Ziegner, Grit Brauckmann, Christoph Witthoefft, Paul Bubenheim, Thomas Waluga

**Affiliations:** 1 Institute for Process Systems Engineering, 38987 Hamburg University of Technology, Am Schwarzenberg Campus 4, Hamburg 21073, Germany; 2 Institute of Technical Biocatalysis, 38987 Hamburg University of Technology, Denickestraße 15, Hamburg 21073, Germany

**Keywords:** multienzymatic cascade, model development, model validation, biocatalysis, miniplant, enzyme membrane reactor, reactive extraction centrifuge

## Abstract

The development and validation of a mathematical model for a highly integrated biocatalytic process are presented. A multienzymatic cascade comprising three parallel and sequential reaction steps was implemented in a miniplant setup, integrating an enzyme membrane reactor (EMR) and a reactive extraction centrifuge (REC) to enable intensified biotransformations. Laboratory-scale experiments were conducted to obtain the kinetic and thermodynamic data necessary for model development. The resulting process model captures both the enzymatic reaction kinetics and the dynamic behavior of the integrated unit operations. Model validation was performed under representative process conditions in the miniplant system. The results demonstrate good agreement between the model and experimental data, confirming the predictive power of the model and supporting its use in future process design, scale-up, and optimization. This work underscores the importance of model-based development for advancing sustainable and efficient biocatalytic processes.

## Graphical abstract

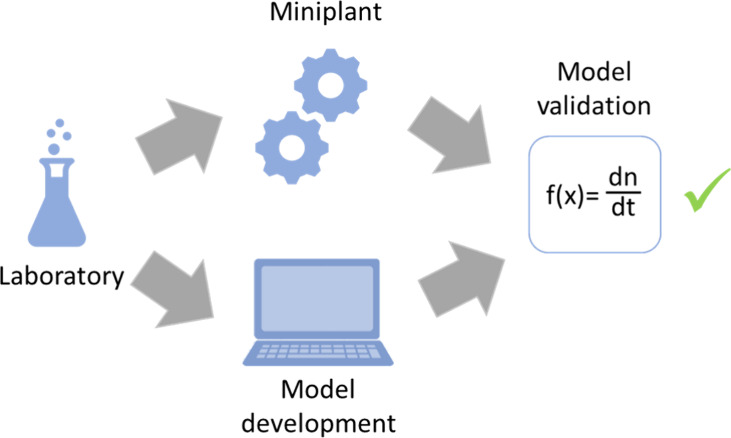

## 1 Introduction

### 1.1 Motivation

Biocatalysis offers a sustainable alternative to conventional chemical synthesis by enabling reactions under mild conditions, with high (stereo)­selectivity, reduced environmental footprint, and often lower energy requirements.^1–3^ Enzymes, as biocatalysts, are capable of transforming complex molecules with high specificity, making them attractive for applications in pharmaceuticals and fine chemicals.^2,4,5^ Multienzymatic cascade reactions, in particular, promise process intensification by enabling multistep transformations in a single operational sequence, thereby reducing intermediate concentration and interim purification and improving the yield.^6^

Despite these advantages, several challenges continue to hinder the broader industrial application of biocatalytic processes, particularly for multienzymatic cascades carried out in vitro. There are limitations in enzyme stability, limited process windows because of different required conditions for different enzymes, suboptimal product yields due to equilibrium constraints, diluted solution, and difficulties in downstream processing.^1,3,7–9^ These hurdles often result in insufficient economic competitiveness compared with traditional chemical routes. Overcoming these barriers requires not only process intensification but also a deep understanding of the process, which can be achieved through mathematical modeling.^10^ Model-based development is a crucial tool for the design, scale-up, and optimization of biocatalytic processes.^4,11,12^ The mathematical model enables the prediction of system behavior under varying conditions, conducting sensitivity analyses and developing process intensification ideas.^13–16^ One way of intensifying processes is through process integration.^17^ This involves combining two unit operations into a single device. In biotechnology, this is often referred to as in situ product removal.^18^ In principle, however, even more basic operations can be combined, resulting in highly integrated devices.^19^ Once a mathematical model is developed, it can assist in scaling and optimizing these complex integrated processes. For integrated systems, mathematical models offer the ability to capture the interconnection between the individual steps and identify the bottleneck. Therefore, validation of the developed model is essential to ensure its reliability and applicability for the process. In the context of biocatalysis, model validation offers further challenges due to the complexity of enzymatic mechanisms, enzyme deactivation, and the influence of multiphase interactions.^13^ This requires additional studies on a laboratory scale to identify kinetic parameters and substance-related parameters.^20^

Process integration is a well-known approach to enhance the efficiency of biocatalytic processes, especially in whole cell biocatalysis.^21,22^ In the case of inhibition of the enzymes and/or the occurrence of equilibrium-limited reaction steps, integrated process designs that combine reaction and separation are especially beneficial.^23^ For example, coupling enzymatic reactions with in situ product removal techniques—such as reactive extraction or membrane separation—can shift equilibria, prevent product inhibition, and enhance overall conversion.^10^ Moreover, integrated systems minimize the number of unit operations, potentially reduce energy consumption as well as investments, and simplify downstream processing, all of which are crucial for the economic competitiveness of biocatalytic processes.^10,12^ Especially in multienzymatic cascades, where the intermediate concentration is critical, process integration enables high conversions. As such, the development and modeling of integrated processes and process systems with several reactions are essential steps toward the industrial application of biocatalytic processes.^24^

This work presents the development of an experimental setup and a comprehensive process model for a multienzymatic cascade. A highly integrated miniplant, incorporating an enzyme membrane reactor (EMR) and a reactive extraction centrifuge (REC) to enable simultaneous reaction and separation, is constructed and used to validate the model.

### 1.2 Process Description

The biocatalytic process under investigation enables the sustainable synthesis of the natural flavor compound cinnamyl cinnamate via a multienzymatic cascade reaction in a two-phase system and is shown in Figure 1.

**Figure 1:**
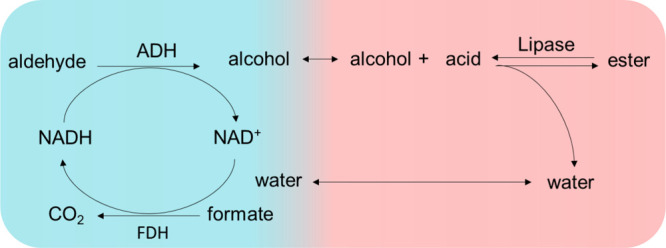
Multienzymatic cascade for the production of cinnamyl cinnamate (ester) with the aqueous phase in blue and organic phase in red (ADH: alcohol dehydrogenase; FDH formate dehydrogenase), adapted from ref 30. Copyright 2025 Chemical Engineering and Processing.

The reaction cascade consists of three reaction steps, two in the aqueous phase and one in the organic phase, coupled by an integrated reactive extraction step. The organic phase is marked red, and the aqueous phase is marked blue in Figure 1. The reaction system has been described before^25–31^ and can be summarized as follows: the initial reaction involves the reduction of cinnamyl aldehyde to cinnamyl alcohol, catalyzed by an alcohol dehydrogenase (ADH) in the presence of the cofactor NADH. Since NADH is consumed stoichiometrically, a formate dehydrogenase (FDH) is employed in a parallel cofactor regeneration step, reducing NAD^+^ back to NADH by oxidizing formate to CO_2_. As both enzymes are sensitive to organic solvents, they are required to be operated in an aqueous phase. The final reaction step, which is the esterification of the intermediate cinnamyl alcohol and cinnamic acid, on the other hand, requires an organic medium to shift the equilibrium to the product site as water is a byproduct. The cascade reactions are linked by an extraction: The aqueous phase and the organic phase are mixed so that the intermediate cinnamyl alcohol can be extracted into the organic phase. These different medium requirements of the three enzymes in the cascade offer additional challenges for the operation on a miniplant scale. In a previous study, these challenges were met by using two reactors, one for each phase and an extractive centrifuge for the mass transfer between both phases.^26^ To retain the enzymes in the corresponding phase, immobilized enzymes were used. In the aqueous phase, the ADH and FDH immobilized on silica particles were retained in a SpinChem reactor. For the organic phase, a fixed-bed reactor filled with Novozyme 435 was used. With this setup, a successful proof of concept was generated.^26^ With a space-time yield of 0.0125 mmol L^–1^ h^–1^, it still offers space for improvement. Simulative studies identified two main bottlenecks: the immobilization of ADH and FDH and the recycling rate between the extractive centrifuge and the fixed-bed reactor in the organic phase.^29^ The immobilization of ADH and FDH led to a significant decrease in the enzyme activity to 4% compared to native enzymes.^28,31^ An option to overcome this is an enzyme membrane reactor (EMR). An EMR retains the enzymes in a specific location by either immobilizing them on the membrane or using a membrane with a cutoff below the enzyme size.^30,32,33,34,33^ The second bottleneck, the recycling rate between the organic phase reactor and the extractive centrifuge, can be overcome by integrating the reactor into the extractive centrifuge,^35^ resulting in a novel reactive extraction centrifuge (REC).^25^

In this study, these two variations are implemented for the first time on a miniplant scale for the production of cinnamyl cinnamate. The detailed setup for the highly integrated miniplant is shown in Figure 2 on the left. In Figure 2 on the right, a photo of the miniplant can be seen to show an impression of the setup. Both dehydrogenase reactions are conducted in an EMR (unit 2 in Figure 2), a pressurized system that retains the freely dissolved enzymes. The EMR^30^ is operated at 35 °C, pH 8.0, and 20 bar. A modular envelope membrane unit (798 cm^2^ membrane area and 1.2 L hold-up) facilitates enzyme retention. A volume flow of 150 mL min^–1^ through the EMR is applied, which equals a residence time of 8 min. A PEBAX on the PAN membrane is used. Circulation is driven by a centrifugal pump to prevent the deposition of particles on the membrane. A piston pump is used to ensure stable operating pressure. The remaining aqueous volume is stored in the heated tank (unit 3 in Figure 2). A total aqueous medium volume of 1.5 L is used. The intermediate cinnamyl alcohol is transferred into the organic phase via the REC (unit 1 in Figure 2). The REC is based on a CINC V02 centrifuge, modified to house immobilized lipase in the rotor zone and protected by a metal mesh. A newly developed rotor with separation and reaction compartments is used.^25^ A volume flow of the aqueous phase of 330 mL min^–1^ and of the organic phase of 430 mL min^–1^ is applied. This leads to a retention time of 0.2 min. The remaining volume of the organic solvent solution is stored in a tank also at 35 °C (unit 4 in Figure 2).

**Figure 2:**
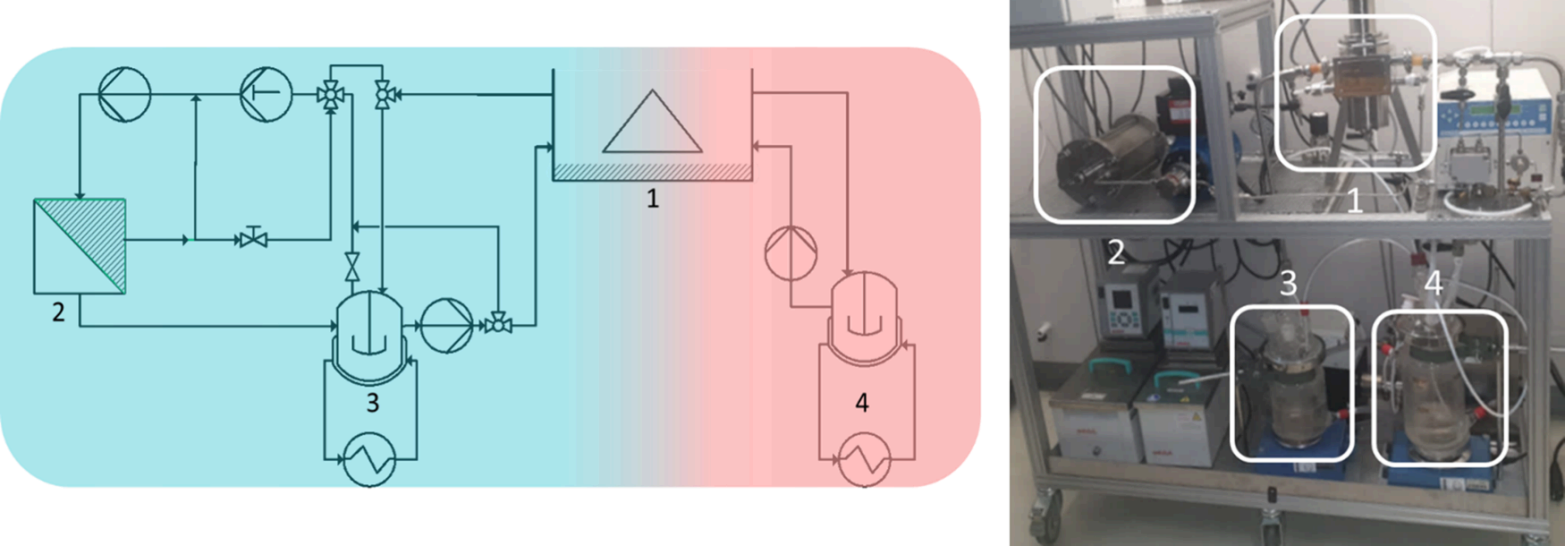
Left: Process scheme of the process for the synthesis cinnamyl cinnamate, shaded area: location of the enzymes with lipase in 1 and dehydrogenases in 2, adapted from ref 30. Copyright 2025 Chemical Engineering and Processing. Right: Photo of the described miniplant, both with unit 1: REC, unit 2: EMR, unit 3: storage tank for the aqueous tank, and unit 4: storage tank for the organic phase.

## 2 Methods

### 2.1 Model Development

To study the given process deeply, a dynamic process model is developed. The model, implemented in Python, is based on ordinary differential equations that describe the molar balances of all components. The molar balances are solved in discrete time steps of 1 min using the odeint solver from scipy. Using Python ensures transparency, reproducibility, and flexibility through open access to model equations and integration with data analysis libraries, enabling a dynamic and reproducible process modeling without licensing restrictions.

The basic structure of the model is shown in Figure 3. As the process is operated below the maximum solubility of all components, precipitation is not expected. The model consists mainly of the volume of the aqueous and the organic phase (units 3 and 4), the EMR (unit 2), and the REC (unit 1). The molar balances of the aqueous and organic phases are implemented according to eqs 1 and 2, respectively. For the aqueous phase in eq 1 *, V̇*_REC_ and *c*_aq,mix_ represent the volume flow and concentration from the REC into the aqueous tank, *V̇*_mem_ and *c*_mem*,i*_ represent the volume flow and concentration from the EMR into the aqueous tank, and *V̇*_aq_ and *c*_aq,*i*_ represent the volume flow and concentration from the aqueous tank into the EMR and REC, respectively. In eq 2, which describes the organic phase, *V̇*_org_ represents the volume flow between the organic tank and REC, *c*_org,mix_ represents the concentration with which the organic phase leaves the REC, and *c*_org_ represents the concentration with which the organic phase leaves the organic phase tank. It is assumed that in the aqueous phase, all components except cinnamyl cinnamate (components *i* are soluble, while in the organic phase, only cinnamyl aldehyde, cinnamyl alcohol, cinnamic acid, and cinnamyl cinnamate, components *j*) are soluble.^26^

$$\frac{dn_{\mathrm{aq},i}}{dt}={\mathrm{V̇}}_{\mathrm{REC}}\cdot c_{\mathrm{aq},\mathrm{mix},i}+{\mathrm{V̇}}_{\mathrm{mem}}\cdot c_{\mathrm{mem},i}-{\mathrm{V̇}}_{\mathrm{aq}}\cdot c_{\mathrm{aq},i}$$ 1

$$\frac{dn_{\mathrm{org},j}}{dt}={\mathrm{V̇}}_{\mathrm{org}}\cdot c_{\mathrm{org},\mathrm{mix},j}-{\mathrm{V̇}}_{\mathrm{org}}\cdot c_{\mathrm{org},j}$$ 2

**Figure 3:**
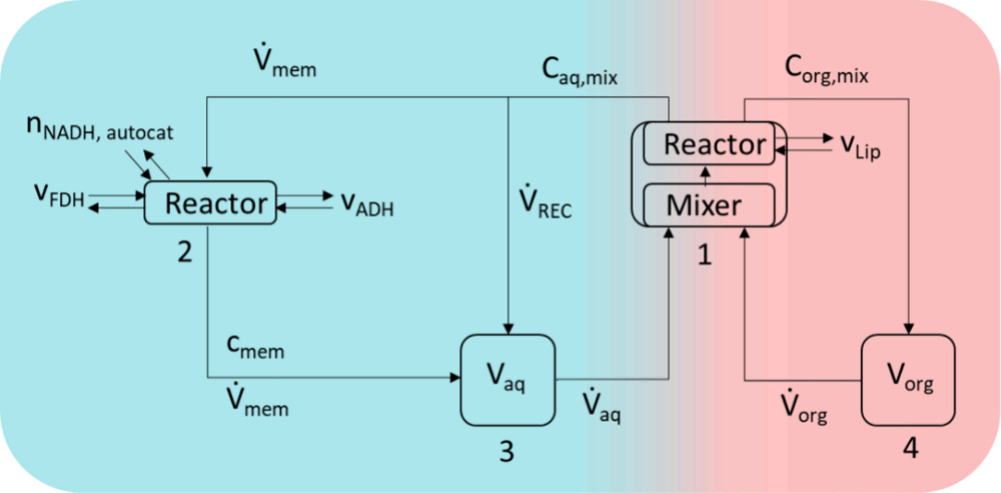
Scheme of the mathematical model for the highly integrated process for the synthesis of cinnamyl cinnamate.

The REC (unit 1) is modeled as a cascade of a mixer and reactor. The phase contact between the organic and aqueous phases is assumed to be sufficiently intense to justify the instantaneous establishment of the phase equilibrium, which is described by component-specific partition coefficients. This assumption is supported by previous detailed investigations of an analogous reactor configuration, in which the characteristic time scales of interphase mass transfer were shown to be significantly shorter than those of the enzymatic reactions.^26^ Under such conditions, mass transfer does not constitute a rate-limiting step. Such simplifications are then applied in reaction kinetics modeling, where processes with markedly different characteristic rates are separated, and fast steps are treated as instantaneous equilibrium.^36–38^ Consequently, interfacial mass transfer resistances are neglected in this work, and phase equilibrium is assumed to be established instantaneously at the REC outlet. The implications and limitations of this assumption are explicitly acknowledged, and the model is intended to capture the dominant kinetic and phase-partitioning effects. For the reactor, an enzyme rate equation *v*_Lip_ is used as a source or sink term depending on the stochiometric factor ν_*j*_. Due to the high-volume flow of 760 mL min^–1^ compared to the hold-up of 150 mL and high rotational speed of 420 rpm, ideal mixing and no concentration gradients are assumed in the mixing and reaction zone. The molar balance for the REC is shown in eq 3, exemplary of component *j* in the organic phase.

$$\frac{dn_{\mathrm{org},\mathrm{REC},j}}{dt}=P_{j}\cdot c_{\mathrm{aq},\mathrm{REC},i}\cdot {\mathrm{V̇}}_{\mathrm{org}}+m_{\mathrm{Lip}}\cdot v_{j}\cdot v_{\mathrm{Lip}}$$ 3

The EMR is modeled as a continuous stirred tank reactor. As with the centrifugal pump, a high-volume flow of 1.5 L min^–1^ over the membrane area of 800 cm^2^ is achieved, and ideal mixing is provided. The reaction rates for the ADH *v*_ADH_- and FDH *v*_FDH_-catalyzed reactions are described with extended Michaelis–Menten kinetics (Section 3.2). Besides the term for the reaction, an autocatalysis of NADH to NAD^+^ is considered, as described in eq 4.^26^ The molar balance for an exemplary component *i* in the EMR is shown in eq 5.

$$\frac{dn_{\mathrm{NADH},\mathrm{autocat}}}{dt}=c_{\mathrm{aq},\mathrm{NADH}}\cdot \left(1-0.66\cdot e^{-0.00197t}\right)$$ 4

$$\frac{dn_{\mathrm{NADH},\mathrm{autocat}}}{dt}={\mathrm{V̇}}_{\mathrm{mem}}\cdot c_{\mathrm{aq},\mathrm{mix},i}-{\mathrm{V̇}}_{\mathrm{org}}\cdot c_{\mathrm{org},j}+m_{\mathrm{ADH}}\cdot v_{i,\mathrm{ADH}}\cdot V_{\mathrm{ADH}}+m_{\mathrm{FDH}}\cdot v_{i,\mathrm{FDH}}\cdot V_{\mathrm{FDH}}$$ 5

### 2.2 Determination of Model Parameters on a Laboratory Scale

#### 2.2.1 Enzyme Kinetics for Lipase

The kinetic parameters of the lipase-catalyzed esterification are obtained from experimental data generated within this work: The experiments are conducted on a laboratory scale in a stirred flask with a total volume of 400 mL. Experiments are carried out in a batch containing a 50:50 vol % mixture of a 0.1 M potassium phosphate buffer and xylene, with Novozym 435 as the immobilized lipase at 35 °C. Reaction progress is monitored for up to 100 h, with samples taken every 30–60 min across 16 experimental runs. The data sets retrieved from these experiments are shown in the Supporting Information.

#### 2.2.2 Partition Coefficient for Cinnamic Acid

The experimental procedure to determine the partition coefficient for cinnamic acid involves mixing 1 mL of 0.1 M potassium phosphate buffer (pH 8.0) with 1 mL of xylene containing cinnamic acid at initial concentrations ranging from 20 to 100 mM in the xylene phase. Each condition is measured in triplicate. After shaking the mixture for 10 min, the residual cinnamic acid concentration in the xylene phase is quantified via gas chromatography.^31^

### 2.3 Operation of Multienzymatic Cascade on a Miniplant Scale

In contrast to the laboratory-scale experiments described above, independent experiments are conducted combining all three reaction steps by a parallel operation of the EMR and REC. Five experiments are conducted for the validation of the model at different operating conditions. The organic phase volume is maintained at 0.6 L, while the aqueous phase volume is 1.5 L. The larger volume of the aqueous phase is due to its presence in both the EMR and the REC, whereas the organic phase is only circulated within the REC. The volumetric flow rates are set as follows: 150 mL min^–1^ for the EMR, 330 mL min^–1^ for the aqueous phase in the REC, and 430 mL min^–1^ for the organic phase in the REC. This leads to a retention time of 8 min in the EMR and 0.2 min in the REC. A stirring speed of 420 rpm is applied in the REC. This combination of flow rates and stirring speed ensures efficient phase separation while minimizing mechanical stress on the enzymes, thereby preventing the destruction of the carrier.^25^ A total of 8.2 g for two experiments and 10.2 g for three experiments of immobilized lipase (Novozym 435) is used. These amounts were kept constant for two and three experiments, respectively, as the enzyme is fixed within the REC and reused across these experiments. The reuse did not lead to a measurable activity loss. The deviation from the mathematical model does not show an increase over the five experiments, which proves this assumption. To rule out enzyme leaching, samples of the experiments are remeasured again over several hours. Since the substrate and product concentrations remain constant, no additional catalytic activity attributable to potential enzyme leaching is detected. The quantities of the native enzymes ADH and FDH range from 6 to 12 and 10 to 20 mg, respectively. These concentrations ensure a reaction process in which none of the enzymes become a significant bottleneck. All experiments are conducted at a temperature of 35 °C. This temperature is selected because it ensures good enzymatic activity for all three enzymes involved without causing denaturation. Samples are taken in 15–30 min intervals. The initial conditions are chosen within the boundaries of the determination of the model parameters. Therefore, it has to be clearly stated that the results from comparing the experimental and simulative results apply for an interpolation within these boundaries and are not suitable for an extrapolation.

## 3 Results and Discussion

### 3.1 Model Parametrization

To enable an accurate description of the multienzymatic cascade, a detailed parametrization of the underlying kinetic and thermodynamic relationships with independent experimental data is essential. Therefore, the determination of the enzyme kinetics and partition coefficients is shown. All parameters applied in the model are determined on the laboratory scale using independent experiments and are not adjusted or refitted using data obtained from the miniplant setup. The miniplant experiments are used solely for model validation, ensuring a clear separation between parameter identification and model verification.

#### 3.1.1 Enzyme Kinetics

For the ADH- and FDH-catalyzed reactions, irreversible Michaelis–Menten kinetics involving two substrates and a product inhibition were applied (eq 6). Temperature-dependent parameters were taken from earlier studies using the Arrhenius approach.^30^ A spline interpolation method is used to determine reaction rates by differentiating the fitted concentration–time profiles, which are subsequently regressed to the kinetic rate equation. This methodology is particularly well suited for complex enzymatic systems and provides robust results with less dependence on initial guess when estimating parameters from progress curves.^39,40^ This leads to maximum reaction rates for ADH of 4.8 mM·min^–1^·mg_ADH_^–1^ and FDH of 5.17 mM·min^–1^·mg_FDH_^–1^. The *K*_m_ values for the ADH-catalyzed reaction are 0.2 and 4.2 mM for NADH and aldehyde, respectively. For the FDH-catalyzed reaction, the *K*_m_ values are 5.3 × 10^–2^ and 112.4 mM for NAD^+^ and formate, respectively. The inhibition constants *K_i_* are 1.5 mM for alcohol in the ADH-catalyzed reaction and 2.7 × 10^–2^ mM for NADH in the FDH-catalyzed reaction.

$$v=v_{\max}\cdot \frac{\left[A\right]\cdot \left[B\right]}{K_{m,A}\cdot K_{m,B}\cdot \left(1+\frac{\left[P\right]}{K_{i,P}}\right)+K_{m,A}\cdot \left[B\right]\cdot \left(1+\frac{\left[P\right]}{K_{i,P}}\right)+K_{m,B}\cdot \left[A\right]+\left[A\right]\cdot \left[B\right]}$$ 6

For ADH, *A* = NADH, *B* = cinnamyl aldehyde, and *P* = cinnamyl alcohol. For FDH, *A* = NAD^+^, *B* = formate, and *P* = NADH

Lipases are well-studied enzymes that typically follow a bi–bi mechanism in which two substrates are converted and two products are released. In the present case, however, water, the product of the esterification reaction, is present in large excess and at constant saturation due to the continuous contact between the organic and aqueous phases in the centrifuge. As a result, water cannot be considered as a reactant in the kinetic description. Its effect on the reaction kinetics manifests as competitive inhibition, which, under isothermal conditions and constant water concentration, can be incorporated into the apparent Michaelis–Menten constants *K*_m,app_ as shown in eq 7. This applies for eq 8, and in the following discussion, for clarity, *K*_m,app_ is abbreviated as *K*_m_. As stated in literature, the reaction medium can have a strong effect on the reaction mechanism itself.^41–43^ Therefore, different possible reaction mechanisms for lipase are possible. For this publication, an ordered bi–uni mechanism^11^ is used, as described in eq 8. It should be emphasized that this reference is used solely to define the mathematical structure of the kinetic model, while all kinetic parameters applied in this study are independently determined.

$$K_{M,\mathrm{app}}=K_{M}\cdot \left(1+\frac{c_{\mathrm{water}}}{K_{i,\mathrm{water}}}\right)$$ 7

$$V_{\mathrm{Lip}}=\frac{V_{\max,f}\cdot (\left[\mathrm{COH}\right]\cdot \left[\mathrm{CAc}\right]-\frac{\left[\mathrm{CCi}\right]}{K_{\mathrm{Eq}}})}{K_{i,\mathrm{CAc}}\cdot K_{m,\mathrm{COH}}+K_{m,\mathrm{COH}}\cdot \left[\mathrm{CAc}\right]+K_{m,\mathrm{CAc}}\cdot \left[\mathrm{COH}\right]+\left[\mathrm{COH}\right]\cdot \left[\mathrm{CAc}\right]+K_{i,\mathrm{CAc}}\cdot K_{m,\mathrm{COH}}\cdot \frac{\left[\mathrm{CCi}\right]}{K_{m,\mathrm{CCi}}}+K_{i,\mathrm{CAc}}\cdot K_{m,\mathrm{COH}}\cdot \frac{\left[\mathrm{CCi}\right]}{K_{m,\mathrm{CCi}}}\cdot \frac{\left[\mathrm{COH}\right]}{K_{i,\mathrm{COH}}}}$$ 8

The time-resolved concentration profiles are evaluated using a spline interpolation-based methodology to extract local reaction rates, which are subsequently used for parameter identification. This approach has been described in detail in previous publications^30,40^ and allows robust determination of kinetic parameters. The methodology for parameter determination is given in the Supporting Information.

The resulting parameters as well as standard errors calculated by the Jacobian matrix calculated with the nonlinear regression are shown in Table 1. The parameters equilibrium constant *K*_Eq_ and the dissociation constant *K*_*i*,COH_ showed higher relative standard errors; this is attributed to their limited occurrence within the rate expression. Both occur only once in the kinetic equation, which reduces the mathematical identifiability. Nevertheless, their magnitudes remain consistent with biocatalytic expectations and studies in that field.^44,45^

**Table 1:** Kinetic Parameter with Standard Errors for the Lipase-Catalyzed Reaction

kinetic parameter	
*v*_max,f_	3.6 × 10^–4^ ± 0.19 × 10^–4^ mM·min^–1^·g_Lipase_^–1^
*K*_Eq_	25.9 ± 1.1 × 10^3^ mmol^–1^·L
*K*_*i*,CAC_	1.1 ± 0.3 mM
*K*_m,COH_	4.5 ± 0.6 mM
*K*_m,CAC_	10.4 ± 2.6 mM
*K*_m,CCI_	0.16 ± 0.04 mM
*K*_*i*,COH_	4.1 ± 5.5 mM

#### 3.1.2 Partition Coefficient

With exception of the cinnamic acid, the partition coefficients for all components are taken from literature.^26^ The partition coefficient for cinnamic acid is experimentally determined due to its expected strong interaction with the buffer system. This interaction is attributed to the deprotonation of cinnamic acid by the phosphate buffer, which is essential for maintaining a constant pH and simulates a proton-removal effect. Since the deprotonated form of cinnamic acid is not detectable with this method and is insoluble in xylene due to its polarity, its presence in the aqueous phase is inferred from mass balance calculations. The results reveal an increasing partition coefficient with higher initial cinnamic acid concentrations. This trend is consistent with the buffer capacity: as the buffer concentration remains constant, a relatively fixed amount of cinnamic acid is deprotonated, while excess cinnamic acid remains in the neutral form that can partition into the organic phase. The observed behavior aligns with the expected thermodynamic equilibrium^46^ and is described by a simple polynomial using eq 9 in the model framework, applying to the studied region of 20–100 mM as the initial concentration.

$$P=0.0002\cdot {c_{\mathrm{org},\mathrm{acid}}}^{2}-0.0002\cdot c_{\mathrm{org},\mathrm{acid}}+0.15$$ 9

### 3.2 Application of the Model on the Experimental Conditions and Comparison of the Results

In this chapter, the experimental results presented in Section 2.3 are compared with the corresponding simulation results obtained from the dynamic process model developed in Section 2.1 and parametrized in Section 3.1. This comparison serves to assess the quality and predictive capability of the developed model under the investigated operating conditions.

The quality of the developed model is assessed by comparing the simulation results to experimental data, as illustrated in Figure 4. The illustrated results of further experiments are given in the Supporting Information. The model used as well as detailed experimental conditions and results for each experiment is given in the repository. The left side of the figure shows the concentration profiles in the aqueous phase, while the right side shows the results for the organic phase. Experimental data points are represented as dots, with the mean deviation from the molar balance over the experiment as error bars. Simulation results are shown as lines. In the aqueous phase, the experimental data show an initial decrease in the aldehyde concentration due to its partitioning into the organic phase. Over the course of the reaction, the expected increase in the alcohol concentration and corresponding decrease in aldehyde were observed. The simulation closely replicates this behavior. In the organic phase, the concentration of the final product, cinnamyl cinnamate, increases steadily, accompanied by the accumulation of the intermediate cinnamyl alcohol. This indicates that lipase-catalyzed esterification is the rate-limiting step of the enzymatic cascade. The second substrate, cinnamic acid, shows an initial rapid decrease, followed by a plateau, consistent with its distribution equilibrium between the two liquid phases and relatively low conversion due to its involvement in only one reaction step. The simulation results agree very well with the experimental data, particularly for cinnamyl cinnamate and cinnamyl alcohol. For cinnamic acid, the simulation slightly overestimates the concentration but still captures its trend accurately.

**Figure 4:**
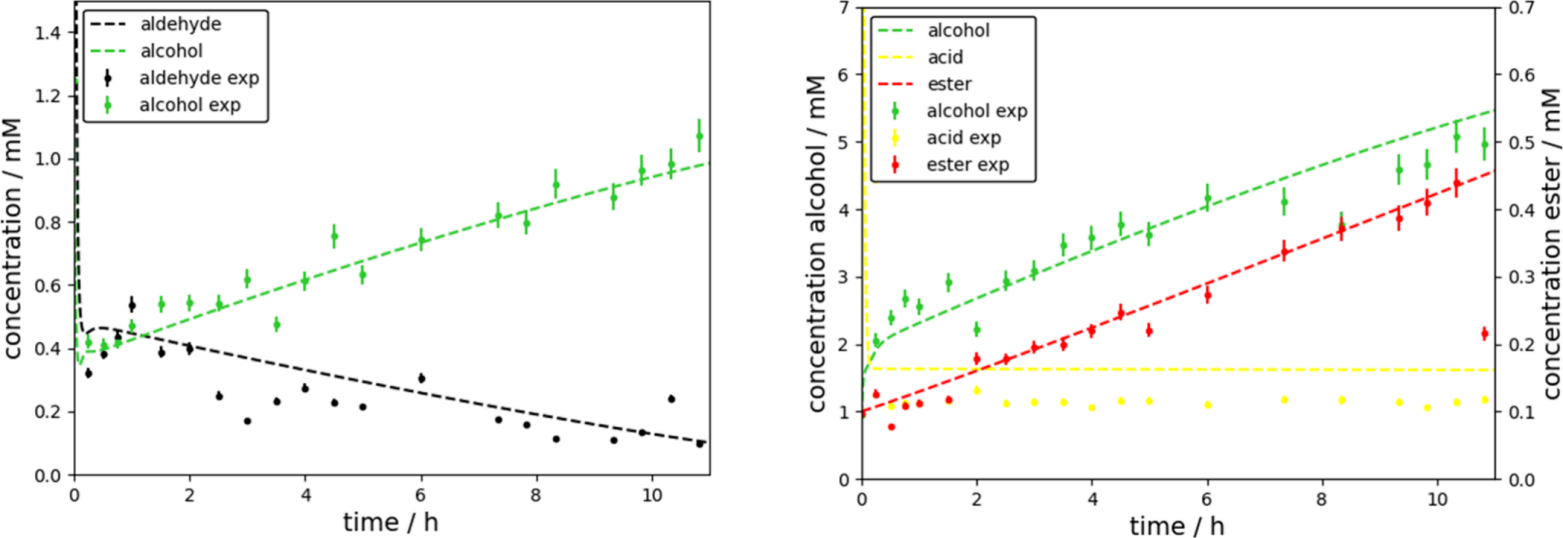
Left: aqueous phase concentrations of cinnamyl alcohol and cinnamyl aldehyde with experimental and simulative results. Right: organic phase concentrations of cinnamyl alcohol, cinnamic acid, and cinnamyl cinnamate with experimental and simulative results, initial conditions: *m*_ADH_ = 10 mg, *m*_FDH_ = 20 mg, *m*_Novozym_ = 10.2 g, *c*_NADH,int,aq_ = 4.9 mmol/L, *c*_Aldehyde,int,aq_ = 6.1 mmol/L, and *c*_Acid,int,org_ = 31 mmol/L.

To determine the component-wise quality of the model, the normalized root mean squared error (nRSME), the coefficient of determination (*R* ^2^), and the mean absolute percentage error (MAPE) are determined. The nRMSE is used to measure how large the prediction errors of the model are relative to the scale of the data. The *R* ^2^ value indicates more generally how well a model explains the variance in the observed data, while MAPE expresses the average prediction error as a percentage of the true values. The results are shown in Table 2. The nRSME shows a particularly strong prediction for cinnamyl alcohol and cinnamyl cinnamate of below 13% in average. The *R* ^2^ and the MAPE show a good match for these components as well. These components are valuable indicators for the model quality, as they are the intermediate and product of the cascade reaction. As described in the model parametrization, the enzyme kinetics were determined for the organic and aqueous phases separately. Within these experiments, the reactions in the two phases are operated in parallel for the first time. Therefore, the good match for the intermediate and product of the enzyme cascade of experimental and simulation data validates the implementation of the reaction kinetics. The highest nRSME is shown for cinnamic acid. This matches the observed systematic deviation from the experimental result and shows the potential for a more detailed study of the partition coefficient for cinnamic acid. This is underlined by the MAPE of 44.9%. This is still an acceptable match but clearly indicates deviations in small concentrations, which are mainly influenced by the partition coefficient.

**Table 2:** nRSME, *R* ^2^, and MAPE for Each Component of the Developed Model

	alcohol aq	aldehyde aq	alcohol org	acid org	ester org
nRSME [%]	12.8	21.0	9.7	33.4	13.8
*R* ^2^	0.899	0.899	0.853	0.944	0.838
MAPE [%]	14.9	14.9	8.0	44.9	13.0

Compared to literature, many biocatalytic models are unfortunately only assessed based on a qualitative match of experimental and simulative results, which applies also for this model.^45,47^ Quantitative analysis of biocatalytic process models remains rare and without a consistent procedure.^13^ Reported deviations between experimental and simulated concentrations range from about 25% for shorter, less complex reaction sequences to above 30% for dynamic simulations.^39,40^ Egger^48^ operated a technical-scale reactive dividing wall column with immobilized Novozym 435; deviations of up to 20% were observed for a single enzymatic reaction. For a single hydrolysis step with independent data, an nRSME of 17% is reported by Lee et al.^49^ Maggi et al.^50^ achieved an nRSME for the model of a fermentation between 3 and 16%. The former publication about the presented multienzymatic cascade with a different setup showed deviations of simulation and experiment between 6 and 26.7%.^26^

As defined by Müller et al.,^51^ nRSMEs of 10–20% for a mechanistic biocatalytic model are satisfactory for model validation. The high *R* ^2^ value of 0.955 demonstrates that the model captures most of the data variance and correctly predicts the general trend of the system. This exceeds significantly the benchmark of 0.75 for satisfying models by Li et al.^52,53^ The MAPE measures the average relative deviation in percentage and is more sensitive to small experimental values, which can lead to the slightly higher error percentages of 23.2 compared to the nRSME. According to the limits defined in literature^53,54^ for models, these values indicate acceptable to good agreement between the model and experimental data. Overall, the results confirm that the model provides an accurate and reliable description of the experimental behavior.

Our study focuses on a model with a primarily mechanistic structure. When machine learning (ML) is used, it is entirely possible to further reduce the deviation between the model and the measured data. Fisher et al.^55^ investigated various ML approaches to predict biotechnological processes. Depending on the ML approach selected, the error ranged between 4 and 13%. Schmitt et al.^56^ used ML to create a model for process control. Here, the error was between 9 and 12%. However, it should be noted that ML approaches generally have significantly more (hyper)­parameters (representing the degrees of freedom) to fit the experimental data, require significantly more data, and do not support a mechanistic understanding of the individual processes. Our results of a mean nRSME of 18.1% between the model and experiment are in line with these literature results, which are in the same magnitude as comparable models and only slightly above machine learning results. Therefore, it can be assumed that the model is suitable to describe the process on the miniplant scale.

## 4 Conclusions

A dynamic process model for a highly integrated multienzymatic cascade reaction is successfully developed and parametrized on a laboratory scale and validated using experimental data from a miniplant setup. The integration of an enzyme membrane reactor (EMR) and a reactive extraction centrifuge (REC) allows for simultaneous reaction and separation, effectively shifting reaction equilibria and improving process performance. The model accurately captures the coupled kinetics and mass transfer phenomena of the three-step cascade across two phases, with an average nRMSE below 20% for the key components, indicating a satisfactory model quality. Particularly strong agreement is observed for the intermediate cinnamyl alcohol and the final product cinnamyl cinnamate, confirming the validity of the implemented enzymatic reaction kinetics and phase interactions. These results demonstrate that the developed model provides a robust foundation for process understanding.

The established model offers a versatile tool for future model-based process optimization, including enzyme amount, initial concentrations, and adjustment of the volume flows to enhance product yield and minimize inhibitory effects. To further improve the model’s predictive accuracy, particularly for cinnamic acid partitioning, the thermodynamic model depth should be expanded. The current empirical approach can be replaced or complemented by physicochemical modeling tools to derive a more accurate partition coefficient for cinnamic acid under process-relevant conditions.^57^ This would reduce the overestimation of the cinnamic acid concentration and, thus, enhance the reliability of the model. Beyond cinnamyl cinnamate synthesis, the modeling framework is adaptable to other multienzymatic or chemoenzymatic processes, particularly those involving phase interfaces and integrated separation. While the current model is based on simplifying assumptions and its predictive testing is limited to the studied system, the underlying structure provides a generalizable approach. Application to different processes requires independent determination of kinetic and thermodynamic parameters and validation under the specific conditions of the new system. Since the reaction mechanism depends on the operating conditions,^41,42^ the question of the appropriate kinetic model must be revisited in this context. Likewise, parameter identifiability is an important issue that needs to be emphasized in future work.^58^ This underscores the broader relevance of the presented approach in advancing sustainable, intensified biocatalytic production platforms.

## Supplementary Material



## Data Availability

The data underlying this study are openly available in TORE (TUHH Open Research) at https://hdl.handle.net/11420/57473

## Notes

A detailed description of the determination of enzyme kinetics using spline interpolation and four additional experimental and simulation data for the operation on the miniplant scale (PDF)
