# Supplementary material for: Development of an Experimental Setup and a Validated Model for a Highly Integrated Biocatalytic Process
Source: Chem Bio Eng. 2026 Mar 26;3(7):641–9. doi: 10.1021/cbe.5c00167 (PMC13403005; doi:10.1021/cbe.5c00167)
Supplement: Supplementary file 1 [file be5c00167_si_001.pdf]

Development of an experimental setup and a validated model for a highly integrated biocatalytic process (Supplementary material)

Francesca von Ziegner[1], Grit Brauckmann[2], Christoph Witthoefft[1], Dr. Paul Bubenheim[2], Dr. Thomas Waluga[1,*]

[1] Institute for Process Systems Engineering, Hamburg University of Technology, Am Schwarzenberg Campus 4, 21073 Hamburg, Germany

[2] Institute of Technical Biocatalysis, Hamburg University of Technology, Denickestraße 15, 21073 Hamburg, Germany

* thomas.waluga@tuhh.de

Table of content

1. Determination of enzyme kinetics using Spline Interpolation..S1
2. Comparison of experimental and simulative data for experiments no.2 – no.5 ....S2

1. Determination of enzyme kinetics using Spline Interpolation

Determination of kinetic parameters, especially for complex enzyme kinetics, is an important topic for modeling biobased process. Direct numerical differentiation of raw experimental data, however, is highly sensitive to measurement noise and therefore unsuitable for reliable rate estimation. For this study a spline-based interpolation is employed to obtain smooth concentration trajectories from the experimental data. A cubic spline interpolation is used to approximate the time evolution of the substrate concentration. The time domain under consideration is divided into *n* intervals using predefined knots, with polynomial functions defined within each interval as specified in Equations (S1) and (S2).

$$c_{S,\,Spline}(t) = \begin{Bmatrix} c_{S,Spline,\,1} \\ \cdots \\ c_{S,Spline,\,n} \end{Bmatrix} \quad \text{(S1)}$$

$$c_{S,\,Spline,i}(t) = a_i \cdot t^3 + b_i \cdot t^2 + c_i \cdot t + d_i \quad \text{(S2)}$$

The substrate concentration $c_{S,\,Spline}$, together with its first and second derivatives, is required to be continuous over the entire time domain. The polynomial coefficients a, b and c define the individual spline segments. The resulting spline functions are twice continuously differentiable, with smooth transitions at the knot points enforced through additional boundary conditions during the fitting procedure. To further ensure a physically meaningful representation of the substrate concentration profile, two additional constraints are imposed: a monotonic decrease of $c_{S,\,Spline}$ with time (Eq. (S3)) and a concave curvature of the concentration profile (Eq. (S4)).

$$\frac{dc_{S,Spline}}{dt} \leq 0 \tag{S3}$$

$$\frac{d^2c_{S,Spline}}{dt^2} \tag{S4}$$

The spline interpolation serves a dual purpose: it reduces experimental noise and provides a differentiable representation of the concentration profiles. This smooth representation can be analytically differentiated to obtain time-dependent reaction rate expressions, which are subsequently evaluated at discrete time points over the investigated time interval. Based on these calculated reaction rates, the parameter estimation is formulated as an algebraic optimization problem, as defined in Equation (S5). The resulting datasets for concentration and reaction rate can then be fitted to the chosen kinetic equation.

$$\frac{dc_{S,Spline}}{dt} = v_{S,Spline}(t) \tag{S5}$$

2. Comparison of experimental and simulative data for experiments no.2 – no.5

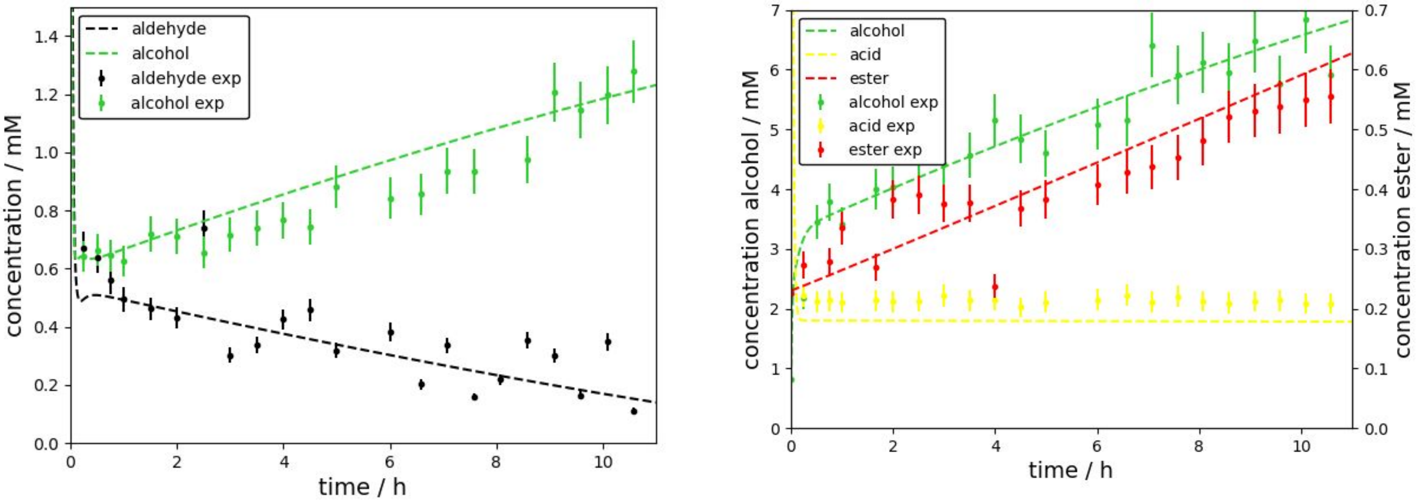

Figure S1: left: aqueous phase concentrations of cinnamyl alcohol and cinnamyl aldehyde with experimental and simulative results, right: organic phase concentrations of cinnamyl alcohol, cinnamic acid and cinnamyl

cinnamate with experimental and simulative results, initial conditions: mADH= 9.9 mg, mFDH= 20 mg, mNovozym=10.2 g, cNADH,int,aq=4.9 mmol/l, cAldehyde,int,aq=6.2 mmol/l, cAcid,int,org=34.7 mmol/l

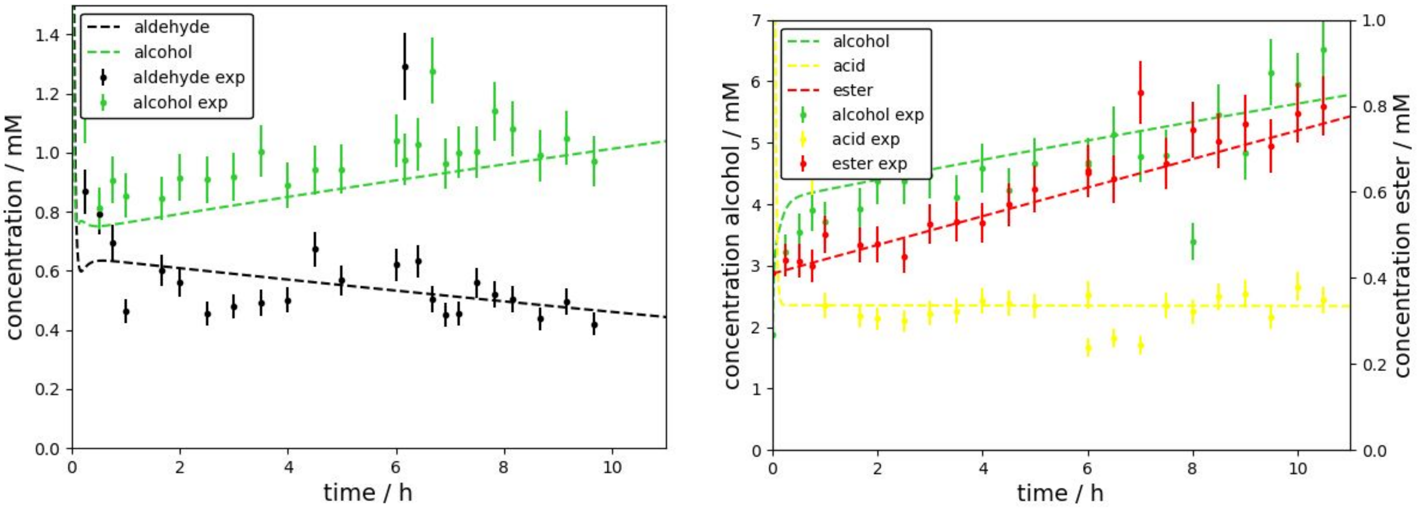

Figure S2: left: aqueous phase concentrations of cinnamyl alcohol and cinnamyl aldehyde with experimental and simulative results, right: organic phase concentrations of cinnamyl alcohol, cinnamic acid and cinnamyl cinnamate with experimental and simulative results, initial conditions: mADH= 6 mg, mFDH= 10 mg, mNovozym=8.2 g, cNADH,int,aq=4.3 mmol/l, cAldehyde,int,aq=6.4 mmol/l, cAcid,int,org=46.7 mmol/l

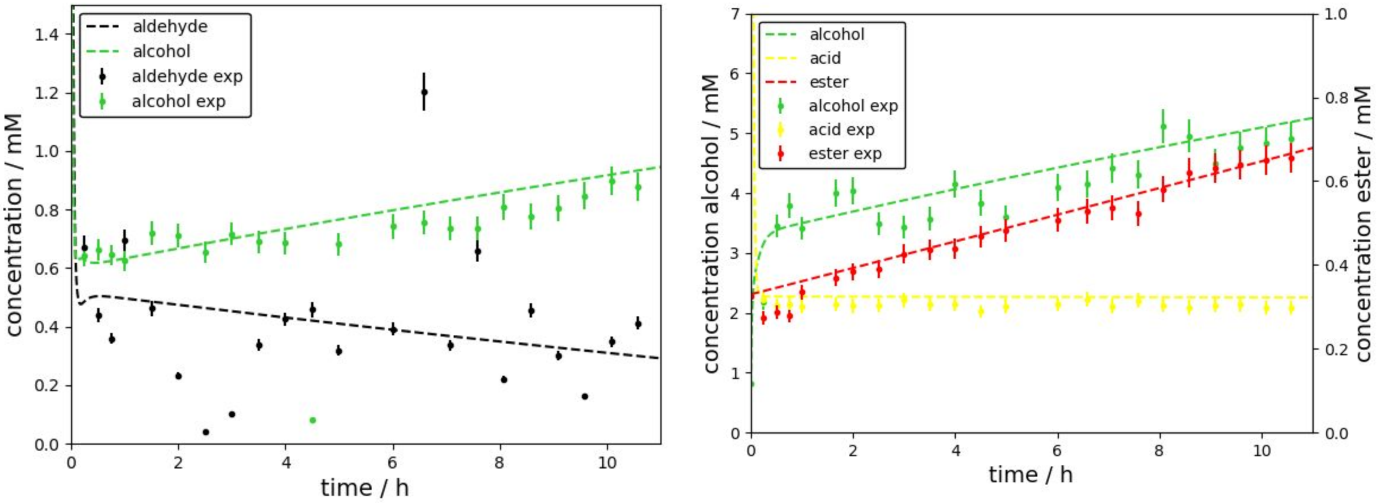

Figure S3: left: aqueous phase concentrations of cinnamyl alcohol and cinnamyl aldehyde with experimental and simulative results, right: organic phase concentrations of cinnamyl alcohol, cinnamic acid and cinnamyl cinnamate with experimental and simulative results, initial conditions: mADH= 10.2 mg, mFDH= 15 mg, mNovozym=8.2 g, cNADH,int,aq=4.3 mmol/l, cAldehyde,int,aq=5.95 mmol/l, cAcid,int,org=44.8 mmol/l

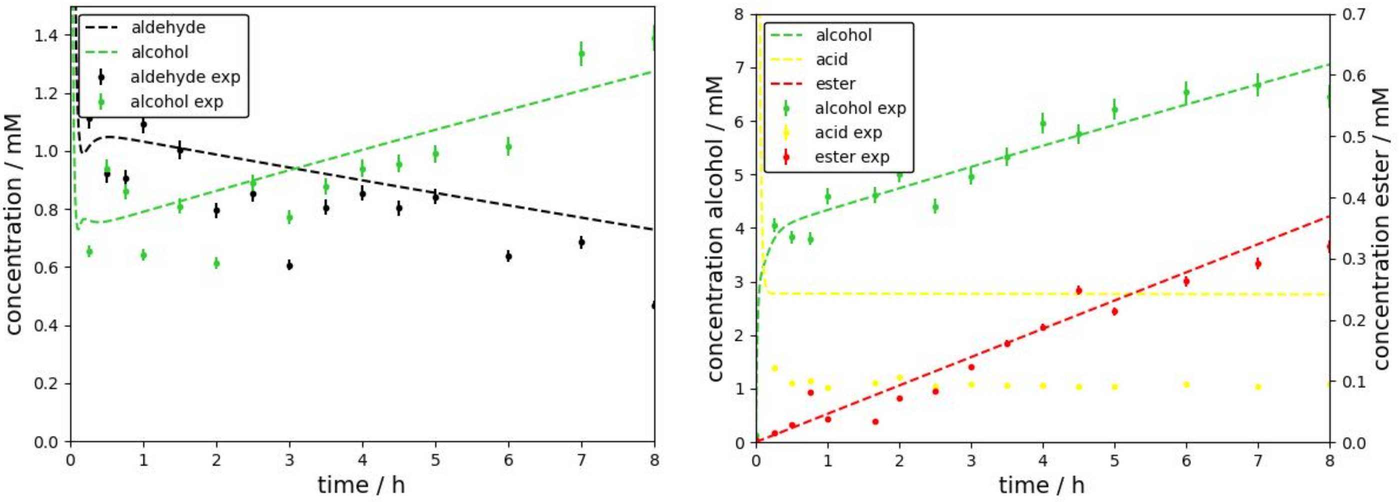

Figure S4: left: aqueous phase concentrations of cinnamyl alcohol and cinnamyl aldehyde with experimental and simulative results, right: organic phase concentrations of cinnamyl alcohol, cinnamic acid and cinnamyl cinnamate with experimental and simulative results, initial conditions: mADH= 12 mg, mFDH= 15 mg, mNovozym=8.2 g, cNADH,int,aq=7.2 mmol/l, cAldehyde,int,aq=12.6 mmol/l, cAcid,int,org=56 mmol/l
